# Mineral Nitrogen Transformation and Microorganism Responses Affected by Organic and Biological Amendment in Intensive Cropping System

**DOI:** 10.3390/plants15162425

**Published:** 2026-08-09

**Authors:** Audrius Jakutis, Regina Skuodienė, Ewald Sieverding, Virgilijus Baliuckas, Jūratė Aleinikovienė

**Affiliations:** 1 Department of Agroecosystems and Soil Science, Agriculture Academy, Vytautas Magnus University, Studentų Str. 11, LT-53361 Akademija, Lithuania; jurate.aleinikoviene@vdu.lt; 2 Bioeconomy Research Institute, Agriculture Academy, Vytautas Magnus University, Studentų Str. 11, LT-53361 Akademija, Lithuania; 3 Vėžaičiai Branch, Lithuanian Research Centre for Agriculture and Forestry, Gargždų Str. 29, LT-96216 Vėžaičiai, Lithuania; regina.skuodiene@lammc.lt; 4 Institute of Agricultural Sciences in the Tropics (Hans-Ruthenberg Institute), University of Hohenheim, Garbenstr. 13, D-70599 Stuttgart, Germany; sieverdinge@aol.com; 5 Institute of Forestry, Lithuanian Research Centre for Agriculture and Forestry, Liepų Str. 1, LT-53101 Kaunas, Lithuania

**Keywords:** intensive cropping system, nitrogen fertilization, biological activator, organic fertilizers, total nitrogen, soil organic carbon, microbial community

## Abstract

Intensive cropping systems often disbalance soil nitrogen (N) cycling due to high mineral fertilization and low organic matter return. We investigated the linkage between soil N transformation and microorganism responses to mineral N fertilization in long-term intensive cropping systems during 2019–2022. A three-factor experiment was established on loam soil, including varying mineral N rates (100, 150, 180, 230 kg N ha^−1^), organic fertilizer (0 or 300 kg ha^−1^), and a biological activator (0 or 0.1 L ha^−1^). Higher mineral N rates combined with organic fertilizer and biological activator significantly increased soil organic carbon and enhanced biologically transformed N (NH_4_^+^ + NO_3_^−^), with the strongest effects observed at N180. Despite increased mineral N inputs, total soil N did not rise, indicating limited long-term N retention. Increasing mineral N inputs reduced total microbial abundance and shifted the community toward bacterial dominance, lowering soil fungi and bacteria ratio and decreasing biological N immobilization. Organic fertilizer inputs counteracted these effects by increasing fungal abundance and improving biological N transformation efficiency. Overall, the study demonstrates that maintaining balanced mineral N inputs together with organic and biological amendments is essential for stable soil N transformation in intensive cropping systems. For sustainable N management, mineral N should not exceed 150 kg N ha^−1^ or 180 kg N ha^−1^, when supplemented with organic fertilizer to avoid soil microbial functioning disturbance and to improve N-use efficiency.

## 1. Introduction

The holistic approach of nitrogen (N) management emphasizes balanced nutrient input into the soil, stabilization of soil biological activity, and ecological feedback for sustained long-term soil productivity [1]. Among these aspects, soil fertility is settled not only as a function of nutrient supply but also as a complex of biological and biochemical processes that regulate nutrient transformation and availability. However, by integrating organic and biological amendments into intensive cropping systems, the natural N cycling can be improved by diverse turnover of soil organic matter, microbial diversity, enzymatic activity, and strengthened soil–plant–microbe interactions [2,3,4]. Even though integrative management practices are already promoted, modern intensive agriculture still relies heavily on mineral N fertilizers to maintain high yields of agricultural plants [5,6,7]. Hence, assessing the interactions between N transformations and microbial community responses under integrative amendment practices is crucial for balancing crop productivity with long-term soil health.

It is evident that mineral N fertilization is still the most widely employed method to maintain high crop yields in intensive cropping systems [8]. The rate of mineral N application depends on crop requirements, soil fertility, climatic conditions, and management practices. In intensive cereal crop systems, mainly 100–300 kg N ha^−1^ yr^−1^ are applied; nevertheless, a sustained high-yield application of N can exceed even 500 kg N ha^−1^ yr^−1^ [9,10,11]. High mineral N inputs exceed crop demand and lead to low nitrogen use efficiency, typically below 50%, with the remaining N lost through leaching, volatilization, or denitrification [12,13]. Imbalanced N fertilization not only represents an economic inefficiency but also contributes to environmental problems, including nitrate pollution of groundwater and emission of nitrous oxide, a potent greenhouse gas [14,15,16]. Consequently, optimizing mineral N application rates according to site-specific soil and crop conditions, while integrating organic and biological amendment, has become a key focus for improving nutrient use efficiency and minimizing ecological risks in intensive agricultural systems.

The application of mineral N fertilizers significantly influences soil chemical and physical properties; hence, long-term N fertilization often leads to negative changes, such as nutrient solubility and imbalance, soil acidification, and soil structure deterioration [17,18,19]. High N application rates are commonly associated with deficiencies in phosphorus, calcium, and magnesium, as soil acidification and nutrient competition reduce nutrient availability to plants [20,21,22]. Additionally, high N application rates disturb the balance of stabilized organic matter, thereby negatively affecting soil structure and cation exchange capacity [22,23]. These alterations reduce soil fertility and limit soil to sustain productive cropping systems in the long-term perspective [24,25].

Higher mineral N inputs influence the structure and function of agricultural soil microbial communities [26]. As high mineral N inputs suppress symbiotic and free-living N fixers (e.g., *Rhizobium*, *Azotobacter* spp.), they also suppress arbuscular mycorrhizal fungi (e.g., *Rhizophagus*, *Claroideoglomus* spp.) and reduce nutrient cycling and soil–plant symbiosis efficiency [26,27,28]. Continuous high N application can alter soil enzyme activities related to carbon and phosphorus cycling and accelerate soil acidification, both of which negatively affect microbial balance and soil health [29,30,31]. Consequently, while high N rates may initially enhance plant growth, they can lead to long-term declines in soil microbial diversity, ecosystem stability, and nutrient-use efficiency. Thus, mineral N rate can decrease the production of microbial biomass in the soil with implications for soil carbon storage and plant nutrient uptake [32,33]. However, increased mineral N inputs can enhance crop plant defense activities and may lead to physiological stress and reduced overall productivity [34]. This can indicate changes in symbiotic rhizobia or mycorrhizal fungi reduction, and thus a shift in the microbial community with the decrease in plant–microbial associative and beneficial diversity [35].

Intensive application of mineral N is related to the continuous increase in soil organic matter or the decomposition rate of added organic matter [36], or may even may approach critical soil organic carbon (SOC) depletion in intensive cropping systems [37]. In this case, the addition of organic amendments is required to stabilize the long-term SOC accumulation and increase the mineral nutrient uptake, as well as to promote soil biological properties, especially an increase in the number of N mineralizers and in total microbial biomass [38]. Nevertheless, few studies have been conducted to clarify the integrated impacts of N application and organic and biological amendment on the correlation with soil nitrogen transformation and microbial community composition changes. As indicated by Köninger et al. [39], compared with mineral N application alone, integrated use of mineral N with organic amendment was related to lower N uptake and did not significantly increase in crop yield. Furthermore, soil microbial diversity changed with a beneficiary increase in the mycorrhizal fungi community [39,40]. Thus, the use of mineral N with biological amendment changed the microbial community composition without impacting diversity, but enhanced soil bacteria biomass and complexity [41,42]. Otherwise, mineral N with biological amendment intensified bacterial-released N losses [43]. According to Vogeler et al. [13], mineral N application alone was related to increased post-harvest residual soil nitrate concentration, although mineral N with organic amendment stabilized carbon fractions, increased microbial biomass [44], and improved microbially-mediated N pathways [45]. However, mineral N application with additional biological and organic amendments changed soil microorganism functional groups and increased microbial abundance and enzyme activity [46]. The above studies indicated that the changes in soil N transformation and in soil microbial response due to diverse mineral N fertilization strategies were interactive and complex. As a result, mineral N transformation remains unclear, especially when implementing intensive cropping practices at the farm level. Understanding the strategies for integrated use of N fertilizers with organic and biological amendments may help in managing the optimal balance between mineral and organic/biological inputs for soil nutrient efficiency, soil biological activity, and long-term soil productivity for sustainable management of intensive cropping systems. In this context, the purposes of this research were (1) to investigate the impacts of integrated use of N fertilizers and organic and biological amendments on soil quality variables, including chemical and biological soil parameters; and (2) to determine the optimum mineral N input for field conditions to encourage farmers to reduce mineral N fertilizer use in order to mitigate nutrient use insufficiency. It was hypothesized that a reduction in mineral N inputs, combined with organic and biological amendments, would improve soil nutrient efficiency and biological stabilization.

## 2. Results

### 2.1. Soil Chemical Properties

The soil chemical properties are presented in Table 1. It was estimated that soil pH changed as the experiment was implemented. Even though in the second year of the experiment (in 2020), soil pH had tended to be lower, it increased in the fourth year (in 2022). The pH significantly increased after applying N150 and N230, respectively, in addition to the OF and BA. Thus, soil pH_KCl_ values were stabilized for four years after jointly applying the OF and BA with N100 and N150 (from 7.2 to 7.3), but after applying N180 and N230, soil pH_KCl_ significantly increased (from 6.8 to 7.5).

As indicated in Table 1, the TON content ranged from 0.11 mg kg^−1^ to 0.24 mg kg^−1^. However, it significantly increased with the increase in mineral N rate, especially with N180 and N230, and after jointly applying the OF and BA. However, the lower mineral N rates (N100 and N150), even with the addition of the OF and BA, decreased the TON content over the fourth year of the experiment. 

The C:N ratio in soil changed to a similar extent, from 7.4 to 13.7; however, significant differences in the C:N ratio were estimated, and lower mineral N rates (N100 and N150) were applied jointly with the BA (Table 1). In addition, even the C:N ratio increased with the increase in mineral N rate in the fourth year of the experiment, but the differences between the treatments were not significant.

### 2.2. Soil Biological Properties

Mineral N fertilization and OF and BA application significantly affected soil biological properties in the surface soil (0–20 cm) (Table 2). It was estimated that the increase in the mineral N rates (N180 and N230) decreased the total microbial abundance by 35%. However, after increasing mineral N rates, along with applying the OF and BA, a significant increase in total microbial abundance (TMA varied from 1.6 × 10^6^ to 2.8 × 10^6^ CFU) was estimated.

The TMA also decreased along with the mineral N rate and the additional application of BA (Table 2). The increase in mineral N rate (N180 and N230) negatively affected the fungi:bacteria ratio, decreasing it by two times on average compared to the N100 impact rate. The significantly higher fungi:bacteria ratio was estimated after applying N180 with the OF and BA addition. 

However, the NBIO (NH_4_^+^ + NO_3_^−^) transformation was lower in extent after applying only the mineral N fertilizers (Table 2). The NBIO content increased after applying higher mineral N rates along with the OF addition, or after combining the OF and BA addition.

### 2.3. Relationship Between Applied Fertilization and Soil Properties

**Figure 1:**
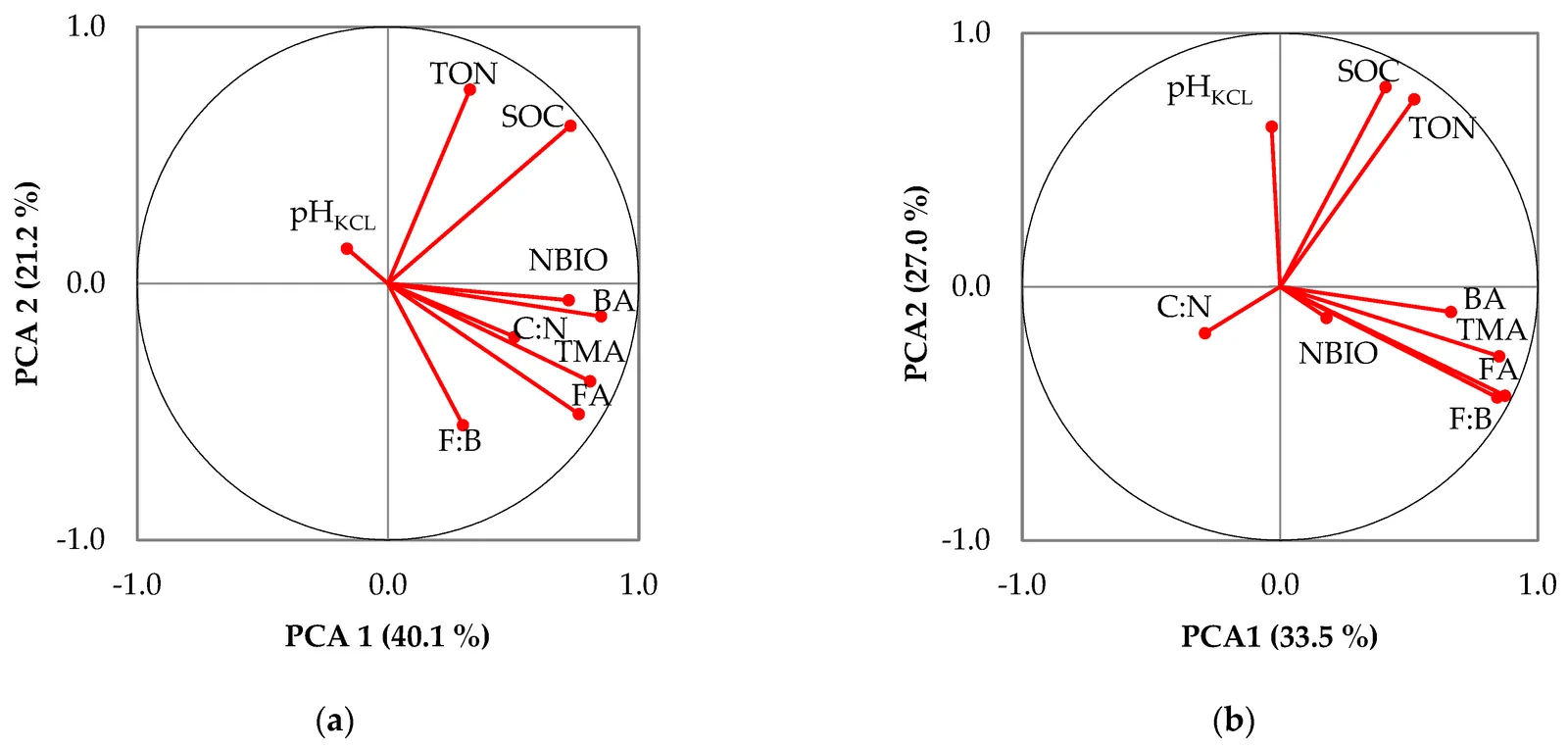
Principal component analysis (PCA) scores and loading biplot showing clustering of soil chemical and biological properties due to the applied fertilization, in order to explain the variation in the response variables (after fertilization in 2020 (**a**) and in 2022 (**b**)). Soil properties variables are indicated with red lines (abbreviations: carbon and nitrogen ratio (C:N), pH value (pH_KCl_), total mineral nitrogen (TON), soil organic carbon (SOC), biologically transformed nitrogen (NBIO), bacteria abundance (BA), fungi abundance (FA), total microbial abundance (TMA), fungi and bacteria ratio (F:B)).

The principal component analysis (PCA) was conducted to analyze the effect of fertilization on soil chemical and biological data dispersion (Figure 1). 

The results showed that data on soil properties dispersion in the second year of the experiment (Figure 1a) in the PC1 accounted for 40.1% of total variance, and in the PC2 it accounted for 21.2% of total variance. Due to the mineral N application and the combination of OF and BA, the higher data dispersion in the PC1 indicated the dominance of soil microbial indicators—the TMA (also separately bacteria (BA) and fungi (FA) abundance), fungi:bacteria ratio, and the NBIO content (Figure 1). However, in the PC2 data of soil chemical properties, mainly TON and SOC were distributed. The distribution of indicators showed that microbiological properties were the most affected and caused distinct shifts in soil properties.

In the fourth year of the experiment, soil chemical and biological data dispersion in the PC1 accounted for 33.5% of total variance, and in the PC2 it accounted for 27.0% of total variance (Figure 1b). Thus, an increase in variance by the PC2 could reflect ongoing long-term fertilization, and could contribute more to the overall relative influence of variability. However, the concentration of soil microbial variables in the PC1 reflected the close relationship between the changing microbial community depending not only on fertilization, but its own effect on nitrogen transformation, and also reflects the changes in soil C:N ratio. The variables of the soil C:N ratio in the PCA components were dynamic (in 2020 and in 2022) and possibly reflect the changes in the SOC and TON.

### 2.4. Study Area Regression Analysis for Soil Variables

**Figure 2:**
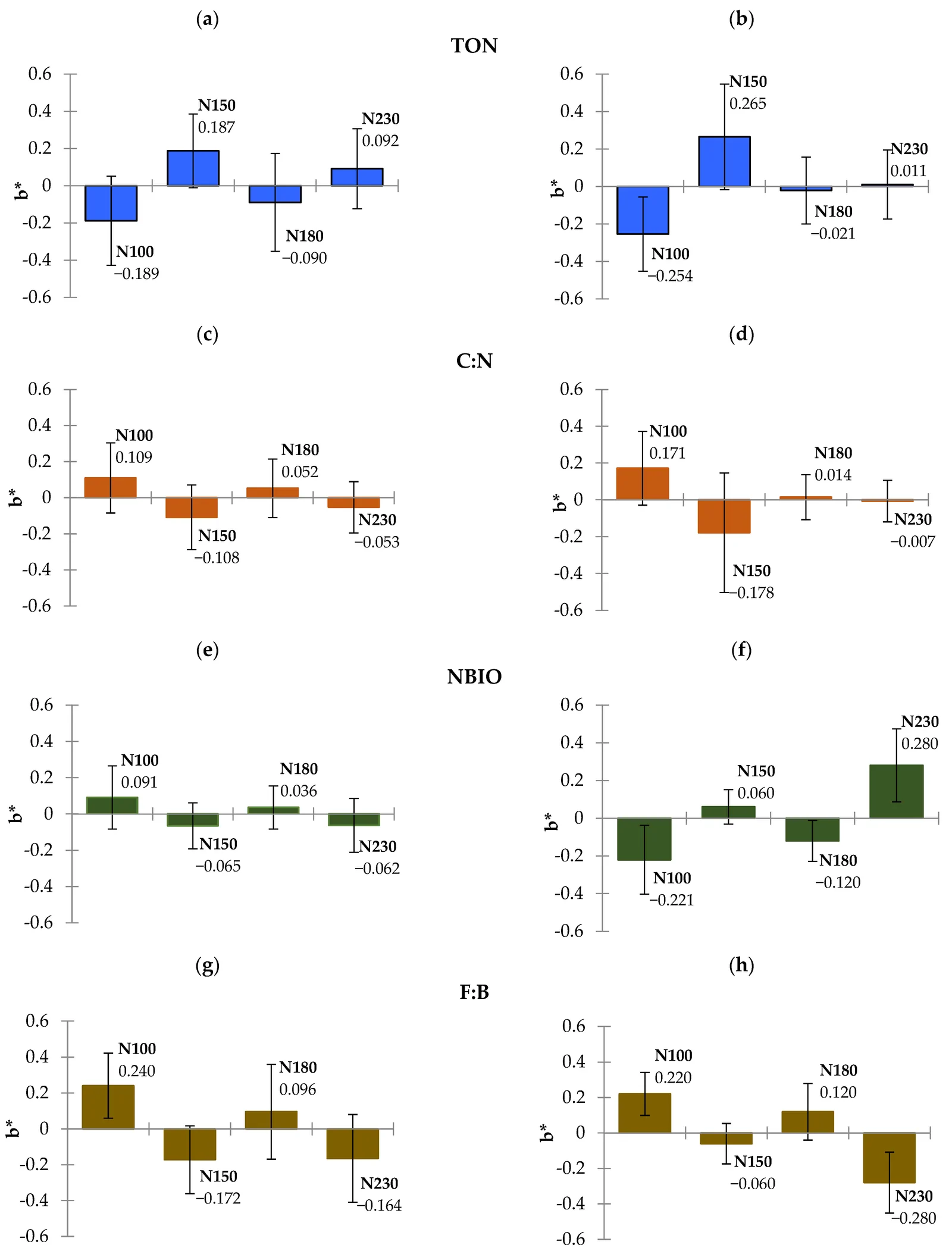
Standardized coefficients in regression (b*) for the soil variables in response to mineral N application. Total mineral nitrogen (TON), carbon and nitrogen ratio (C:N), biologically transformed nitrogen (NBIO), fungi and bacteria ratio (F:B) after fertilization in 2020 (**a**,**c**,**e**,**g**) and in 2022 (**b**,**d**,**f**,**h**). Bars on columns present 95 percent confidence interval range of values calculated from sample data containing the true data set parameter.

In this study, mineral N application and the combination of OF and BA slowed the N release rate and increased N use efficiency. However, mineral N application had the most pronounced impact on soil chemical and biological properties. Consequently, the regression analysis indicated the different variability pathways in soil properties due to the mineral N treatments (Figure 2). It could be noticed that mineral N treatment influenced the variability of TON content (Figure 2a,b). Based on the regression coefficient (b*), the N150 contributed more positively to the variability in TON content (b* = 0.187) in soil in the second year of the experiment. Even in the fourth year, the N150 application strengthened the positive variability of TON content (b* = 0.265) in soil (Figure 2b). In contrast, a lower mineral N application rate (N100) contributed negatively to TON content variability (b* = 0.189 and b* = 0.254). However, the increasing rate of mineral N contributed negatively to, or had a minor impact on, soil TON content variability.

Experimental data on the soil C:N ratio indicated the mineral N availability (Figure 2). However, mineral N application could directly increase crop-derived biomass production, but also indirectly sustain the soil C:N ratio (Figure 2c,d). Based on b*, during the four years of the experiment, only the mineral N100 contributed more positively to the variability in the soil C:N ratio (b* = 0.171; (Figure 2e,f). The increasing rate of mineral N contributed negatively (when N150, b* = −0.178) or had a minor impact on soil C:N ratio variability (b* ≤ 0.052). In this case, it was evident that higher mineral N application altered N pool lability.

Soil biological N immobilization in our experiment did not directly correspond to the mineral N fertilization rate; however, mineral N application changed the composition of the soil microbial community (Figure 2e,f). Taking this into account, it could be noticed that even in the second year of the experiment, soil NBIO transformation variability (b* ≤ 0.091) mostly had a minor impact due to the mineral N application (Figure 2e). However, the mineral N application after four years of the experiment was considered to influence the variability in the soil F:B ratio (Figure 2g,h). A higher mineral N application rate (N230) contributed to a lower soil F:B ratio (b* = −0.280); thus, in this case, the dominance of the bacterial community could increase the ability to intensify the immobilization of mineral N (Figure 2f). In contrast, lower mineral N application rates, N100 and N180, contributed more positively to the variability in the soil F:B ratio (b* = 0.220 and b* = 0.120), but negatively to the variability in soil NBIO transformation (b* = −0.221 and b* = −0.120; Figure 2f). In these cases, it is likely that the microbial community reduced the intensity of mineral nitrogen immobilization. However, N150 application had a minor impact on the soil F:B ratio and NBIO transformation variability. In fact, N150 application did not strongly alter the soil microbial community structure or soil biological N immobilization.

## 3. Discussion

It is evident that mineral N application changes soil chemical and biological properties [19,24,30]. Even after the mineral N is applied, soil nutrient balance should be maintained and soil biological functions should be preserved [47,48]. In this study, mineral N management was selected as factor for indicating soil stability through mineral N transformation (Table 1) and microbial community change (Table 2).

In general, N transformation is expected to be the interaction of the N mineralization process in soil with N mobilization and immobilization within the soil–plant system [49,50] or, on the other hand, with the increase in capacity of N retention in the microbial biomass [32,33]. According to the results of our experiment, N mobilization in soil decreased along with the increased mineral N application rate (Table 1). However, changes in microbial biomass and community depended significantly on mineral N fertilization and supplementary OF application every year (Table 3). In other words, intensive crop production systems require continuous organic matter inputs to support the soil microbial community: first, to obtain organic N for biomass formation and, second, for mineral N release capability during decomposition [51,52].

Higher mineral N application is often found to change microbial structure and N transformation functional genes, and sometimes even microbial diversity [53,54,55]. Based on our results, the increase in mineral N application (especially when applying N180 and N230) decreased the TMA and F:B ratio (Table 2). According to Grzyb et al. [53] and Khmelevtsova et al. [56], higher mineral N rates change the composition of the soil microbial community to fast mineral N assimilating bacteria, but with a lower abundance of capable symbiotic microbial communities (Table 3).

Bacteria dominance in soil accelerates mineral N turnover and increases N availability, however only for a short period, as mineral N is held for a shorter time in bacteria biomass. In this way, long-term nitrogen retention becomes impossible [57,58]. These changes indicate that while mineral N increases short-term N availability, excessive inputs interrupt the balance between microbial community structure and soil biological functioning [59,60]. 

It was estimated that the TON content in soil significantly increased with the increase in the mineral N rate, and with the joint application of the OF and BA (Table 1). However, the lower mineral N rate, even with addition of the OF and BA, decreased the TON content over the fourth year of the experiment. These findings explain the importance of soil enrichment in organic matter along with the increased of the mineral N fertilization rate in an intensive cropping system. Similar estimations were found in several long-term field experiments [61,62,63]. However, there is evidence that even with the addition of the OF, mineral N application rates have limits [64,65]. 

Mineral N transformation without intensified nitrate leaching from mineral fertilizers or organic residues was found to be about 150–200 kg N ha^−1^ with N input [66,67,68,69]. In some soils with a potential for mineral N mineralization, nitrate leaching increased exponentially with an increase in the mineral N application rate, ranging from 120 to 160 kg N ha^−1^ [70,71]. Based on our results, it could be predicted that mineral N application should not exceed a rate of 150 kg N ha^−1^ (Table 2). In addition, based on the F:B ratio (Figure 2g,h), NBIO transformation (Figure 2e,f), and mineral N (Figure 2c,d) variability data, the potential increase in N uptake and N use efficiency (in TON content (Figure 2a,b)) in soil was determined.

With increasing mineral N application, carbon availability and microbial activity becomes limited and leads to reduced efficiency of NBIO transformations [62,65,72]. As estimated in our study, the NBIO transformation decreased after applying only the mineral N fertilizers (Table 2). However, the NBIO content significantly increased after applying mineral N (particularly applying N180) along with the OF addition (Table 3). It is well known that mineral N application changes NBIO transformation through settlement of N immobilization, mineralization, nitrification, or denitrification in soil [49,73]. Thus, mineral N transformation used to be balanced in soils between N immobilization and mineralization [53]. With a higher mineral N rate, the soil reduces the mineral N transformation balance, as the N transformation could not be biologically regulated [74].

Unlike mineral N fertilizers, BA aims to stimulate natural soil microbiological processes without any fertilization effect [75]. According to García-Martínez et al. [76] and Hellequin et al. [77], BA induced significant changes in the composition of active bacterial and fungi communities without impact on mineral N transformation. However, according to Li et al. [50], the BA combined with mineral N fertilizers increased mineral N transformation. The BA that was used in our study activated soil bacteria communities; however, the increase in TON content was not significantly stimulated (Table 1). The use of BA with OF increased mineral N availability on a higher mineral N application rate (N230, Table 1).

## 4. Materials and Methods

### 4.1. Study Area

The experiment was conducted from 2019 to 2022 in an intensive cropping system experimental site located in the south-central part of Lithuania (54°52′ N lat., 23°49′ E long.), at an altitude of 68–72 m above the sea level. The characteristic climate in the location is humid continental, marked by warm summers and cold winters (Table 4). 

The experimental site area was characterized by the presence of Luvisols [78], developed on loam texture soil with chemically rich limnoglacial parent material. The soil texture and main chemical properties were analyzed at the beginning of the experiment ([79]; Table 4). 

An intensive cropping system with long-term winter wheat–winter rapeseed cultivation under conventional tillage began more than ten years before the start of the experiment (Table 5). The study site in the intensive cropping system field was hosted by the farmer. The farmer was not offered any direct payments for the hosting experiment.

Since 2012, long-term soil productivity in the study area has been conserved by adjusting crop residue management practices. An average of 5.4–9.0 t ha^−1^ of straw residues were retained in the site soil by shallow loosening (up to 10 cm depth), and deep loosening (up to 30 cm depth) was carried out prior to winter rapeseed sowing only.

### 4.2. Experimental Design

A long-term on-farm field experiment was established and maintained throughout the study period. A three-factor experiment was established in a randomized complete block design with three replications (48 experimental plots in total). The area of a single experimental plot was 900 m^2^ (30 m × 30 m). The experiment factors included the following:

Factor A—mineral N fertilizer application (100, 150, 180, and 230 kg N ha^−1^; indicated in text as N100, N150, N180, and N230). The corresponding experiment mineral N rates (L ha^−1^) of urea and ammonium nitrate solution (32% N, density 1.3 kg m^−3^) were applied when vegetation started.

Factor B—application of organic fertilizers (0 and 300 kg ha^−1^; indicated OF in text as O−and O+. Organic fertilizers in the form of granulated poultry manure (pH_H2O_ 7.2) contained about 32% organic carbon, 3.6% nitrogen, 3.6% phosphorus, and 2.9% potassium, and were applied (300 kg ha^−1^ of physical weight with 64% of organic matter) in the summer on stubble after harvest.

Factor C—application of biological activator (0 and 0.1 L ha^−1^; with spraying volume of 200 L ha^−1^; indicated BA in text as B− and B+). The biological activator contained bacteria (*Bacilllus megaterium* L. and *Bacilllus subtilis* L. (≥1 × 10^9^ colony forming units ml^−1^) and filamentous fungi (*Trihoderma reesei* L., *Trihoderma longibrachiatum* L. and *Trihoderma asperellum* L. (≥1 × 10^7^ colony forming units ml^−1^) strains, and was applied (0.1 L ha^−1^) in the summer on stubble after harvest.

### 4.3. Sampling and Analysis

In the spring of each year of the experiment before the application of the first mineral N fertilizers, three mineral soil samples (at a depth of 0–20 cm) were composed from the randomly collected 10 subsamples at the location of each experiment plot. A total of 48 soil samples were collected per experiment year. These samples were combined and sieved through a 5 mm mesh sieve to eliminate visible plant debris and stones. A 0.2 mm sieve was used to separate the soil samples in order to analyze the soil organic carbon content. One part of the soil sample was air-dried for the pH, total nitrogen, and soil organic carbon content analysis, while the other was refrigerated at 4 °C for the estimation of biologically transformed nitrogen (NH_4_–N + NO_3_–N) content and assessment of soil microbial community counts.

Soil chemical investigations were performed in the Chemical Research Laboratory of the Lithuanian Research Centre for Agriculture and Forestry. Using an IONLAB pH meter, the pH_KCl_ of the soil was measured in accordance with the ISO 10390:2021 standard [80] (soil–solution ratio 1:2.5). The Kjeldahl method was used to quantify the total nitrogen (TON) in the soil. The method applied for determining the soil organic carbon content (SOC) was ISO 10694:1995 [81]. Biologically transformed nitrogen content and soil microbial community counts were estimated in the Agrobiology Laboratory of Vytautas Magnus University Agriculture Academy. The colorimetric method was used to analyze biologically transformed nitrogen (NBIO; NH_4_–N + NO_3_–N) content in accordance with the ISO 14256-2:2005 standard [82]. Th soil microbial community (bacteria and microscopic fungi) was cultivated using the soil suspension dilution and plate-count technique with selective media: meat–peptone agar and starch–ammonia agar for bacteria using the mineral source of nitrogen [83], and potato dextrose for filamentous fungi and yeasts/yeast-like fungi [84]. All media were autoclaved at 121 °C for 15 min before use. Soil microbial community cultivations were conducted under aerobic conditions at 30 °C for one week. Total microbial abundance (TMA) in colony forming units (CFU) was calculated per gram of dry soil. Bacteria and fungi colony counts were done separately. The fungi:bacteria ratio was calculated based on bacteria and fungi CFU counts. The total microbial abundance was estimated as the sum of fungal and bacterial CFUs.

### 4.4. Statistical Analysis

Research data was statistically evaluated by applying the method of variance analysis of quantitative characteristics using the statistical program SAS (version 9.4). Primary analysis of all variables was performed using the principal component analysis procedure (PCA, XLSTAT statistical program). The Shapiro–Wilk test was performed to test the normality of the data set. There were no or only minor deviations from normal distribution. The *t*-test procedure was applied to pairwise comparisons of experimental treatments (SAS program), which involves checking the equality of variances of two groups before comparing their means. Analysis of variance was used to assess the influence of treatments (year, mineral fertilizers, organic fertilizers, biological activators) and their interactions on the studied soil properties (GLM procedure). Since the biological activator did not have a significant effect on the studied traits, it was not included in the analysis of variance model. Differences between mineral N application rate, organic fertilizer, and biological activator treatments were determined by Tukey’s test at a significance level of *p* ≤ 0.05 (SAS). The results of the study are presented as means ± standard error. The Partial Least Square (PLS) model method was used to evaluate the dependence of soil properties on the year, fertilization rate, organic fertilizer, and biological activator treatments (XLSTAT). Variance analysis was performed with the SAS GLM procedure. 

## 5. Conclusions

It could be concluded that in an intensive cropping system, mineral N transformation occurred due to stabilized soil microbial community composition and activity. Soil microbial composition remained stabilized only when the TMA and F:B ratio increased, and mineral N was immobilized when the NBIO content was higher. Thus, NBIO increased with lower mineral N application rates, and when mineral N application was done in combination with OF. The mineral N application in combination with OF and BA increased the NBIO content only with the higher rate of mineral N; however, it did not increase the TON content or N use efficiency. Mineral N application should not exceed 150 kg N ha^−1^ and may reach 180 kg N ha^−1^ when combined with organic fertilizer, without causing substantial disturbance to soil microbial functioning. Thus, an increasing mineral N application rate increases the probability of disturbing the soil microbial community and soil biological functions. Further research is required to estimate the effectiveness of mineral N application under certain conditions, such as soil type and properties, intensive cropping system crop rotations, and the type of on-farm research-based experiments.

## Figures and Tables

**Table 1:** Soil chemical properties of the 0–20 cm soil layer after fertilization in 2020 and in 2022.

Fertilization	Treatments	pH_KCl_		Total Nitrogen Content (mg kg^−1^)		C:N Ratio	
		Year 2	∆	Year 2	∆	Year 2	∆
N100	B−_O−	**6.8 ± 0.00 d**	+0.3 c	**0.11 ± 0.004 d**	+0.02 c	**13.7 ± 1.03 a**	+1.6 a
	B−_O+	**7.3 ± 0.00 a**	+0.1 ab	0.19 ± 0.009 abc	−0.01 bc	10.1 ± 1.33 abc	+1.5 a
	B+_O−	7.0 ± 0.00 c	+0.3 bc	0.18 ± 0.012 abcd	−0.04 c	**7.5 ± 0.68 c**	+5.0 a
	B+_O+	7.2 ± 0.00 b	0 bc	0.17 ± 0.011 abcd	−0.05 c	9.5 ± 0.48 abc	+2.1 a
N150	B−_O−	**6.8 ± 0.00 d**	+0.6 ab	0.21 ± 0.015 abc	+0.01 abc	9.6 ± 0.63 abc	+1.8 a
	B−_O+	**7.3 ± 0.00 a**	+0.4 a	0.20 ± 0.031 abc	+0.09 a	9.8 ± 1.07 abc	−4.2 b
	B+_O−	7.0 ± 0.00 c	+0.5 ab	0.20 ± 0.017 abc	−0.04 bc	**7.4 ± 1.13 c**	+5.5 a
	B+_O+	7.2 ± 0.00 b	+0.1 bc	0.18 ± 0.007 abcd	−0.02 bc	9.9 ± 1.37 abc	+2.1 a
N180	B−_O−	7.2 ± 0.00 b	+0.1 bc	0.17 ± 0.018 abcd	−0.01 bc	8.8 ± 0.75 abc	+2.1 a
	B−_O+	7.0 ± 0.00 c	+0.3 bc	0.14 ± 0.009 cd	+0.03 bc	10.8 ± 0.22 abc	+1.0 a
	B+_O−	**7.3 ± 0.00 a**	+0.2 ab	**0.24 ± 0.020 a**	−0.07 bc	7.8 ± 0.44 bc	+5.2 a
	B+_O+	**6.8 ± 0.00 d**	+0.5 bc	0.15 ± 0.014 abcd	+0.06 abc	12.7 ± 1.48 ab	−1.9 a
N230	B−_O−	7.2 ± 0.00 b	0 bc	0.18 ± 0.010 abcd	−0.04 bc	9.1 ± 0.31 abc	+2.2 a
	B−_O+	7.0 ± 0.00 c	+0.4 ab	0.15 ± 0.006 bcd	0 bc	10.2 ± 0.80 abc	+0.8 a
	B+_O−	**7.3 ± 0.00 a**	+0.2 a	0.20 ± 0.024 abc	−0.02 bc	9.9 ± 1.54 abc	+2.0 a
	B+_O+	**6.8 ± 0.00 d**	+0.7 ab	0.23 ± 0.007 abcd	+0.01 ab	8.7 ± 0.93 abc	+2.3 a

Note: highest and lowest values are in bold. Different lowercase letters represent significant differences (*p* ≤ 0.05). Values are mean ± SE (*n* = 3). ∆—presents the differences in data between second (2020) and fourth (2022) year data.

**Table 2:** Soil biological properties of the 0–20 cm soil layer after fertilization in 2020 and in 2022.

Fertilization	Treatments	Soil Total Microbial Abundance, ×10^6^ CFU		Fungi:Bacteria Ratio		Biological Nitrogen (NH_4_^+^ + NO_3_^−^) (mg kg^−1^)	
		Year 2	∆	Year 2	∆	Year 2	∆
N100	B−_O−	2.0 ± 0.04 cd	0 bcd	0.018 ± 0.0018 abcd	+0.012 b	114 ± 4.3 efg	+3 ef
	B−_O+	2.0 ± 0.01 cd	+0.4 abc	0.020 ± 0.0007 abc	+0.012 b	174 ± 10.4 ab	−53 def
	B+_O−	2.0 ± 0.03 cd	−0.7 e	0.015 ± 0.0009 def	+0.005 cde	161 ± 4.6 abcd	−43 cdef
	B+_O+	1.8 ± 0.03 de	+0.8 ab	0.015 ± 0.0006 de	+0.014 bc	122 ± 16.5 cdefg	−2 def
N150	B−_O−	1.7 ± 0.05 ef	−0.1 de	0.010 ± 0.0009 fg	+0.004 e	118 ± 10.7 defg	+2 def
	B−_O+	1.9 ± 0.02 d	+0.5 abc	0.016 ± 0.0006 bcd	+0.009 bcd	117 ± 6.7 defg	+16 abcde
	B+_O−	**1.6 ± 0.06 f**	0 de	0.011 ± 0.0007 ef	+0.004 e	137 ± 4.1 bcdefg	−16 def
	B+_O+	**2.4 ± 0.06 a**	+0.4 a	0.014 ± 0.0003 def	+0.018 b	166 ± 6.7 abc	−21 abc
N180	B−_O−	1.7 ± 0.02 ef	−0.4 e	**0.010 ± 0.0003 g**	+0.009 cde	110 ± 3.6 fg	−1 f
	B−_O+	1.9 ± 0.05 d	+0.8 ab	0.016 ± 0.0009 bcd	+0.016 b	154 ± 3.3 abcdef	−17 abcd
	B+_O−	**1.6 ± 0.05 ef**	−0.2 de	0.016 ± 0.0007 cd	+0.002 de	**108 ± 7.2 g**	+13 def
	B+_O+	2.3 ± 0.04 ab	+0.5 a	**0.021 ± 0.0009 a**	+0.026 a	**183 ± 10.0 a**	−32 a
N230	B−_O−	**1.6 ± 0.02 ef**	−0.3 e	0.010 ± 0.0012 fg	+0.004 e	157 ± 15.1 abcde	−10 ab
	B−_O+	2.1 ± 0.06 bc	−0.3 cde	0.014 ± 0.0007 def	+0.003 de	134 ± 9.4 bcdefg	+16 a
	B+_O−	1.7 ± 0.03 ef	−0.2 de	**0.008 ± 0.0003 g**	+0.006 e	**108 ± 1.5 g**	+24 abcde
	B+_O+	1.7 ± 0.03 ef	+1.0 a	0.020 ± 0.0009 ab	+0.008 bc	124 ± 4.6 cdefg	+4 bcdef

Note: highest and lowest values are in bold. Different lowercase letters represent significant differences (*p* ≤ 0.05). Values are mean ± SE (*n* = 3). ∆—presents the differences in data between second (2020) and fourth (2022) year data.

**Table 3:** Estimates of treatment and experiment year significance on soil properties.

**Treatment/Property**	**pH_KCl_**	**TON**	**C:N**	**TMA**	**F:B**	**NBIO**
N fertilization	*	*	NS	***	***	NS
OF addition	NS	NS	NS	***	***	***
N fertilization X OF addition	**	*	NS	NS	*	***
**Year and Treatment/Property**	**pH_KCl_**	**TON**	**C:N**	**TMA**	**F:B**	**NBIO**
Year	***	***	***	***	***	***
Year X N fertilization	NS	NS	NS	**	**	**
Year X OF addition	NS	NS	*	***	***	NS

Note: treatment and interaction significance on soil properties meaning ***—*p* < 0.001, **—0.001 < *p* < 0.01, *—0.01 < *p* < 0.05. NS—not significant. Abbreviations: soil pH value (pH_KCl_), total mineral nitrogen (TON), carbon and nitrogen ratio (C:N), total microbial abundance (TMA), fungi and bacteria ratio (F:B), biologically transformed nitrogen (NBIO).

**Table 4:** Characteristics of the site climatic and pedological indices.

Indices	Experimental Site	
Climatic indices	Conducting experiment average (SRC 2019–2022)	Long-term average (SRC 1974–2020)
Total annual precipitation, mm	572.3	679.7
Annual mean temperature, °C	8.4	7.5
Growing season total precipitation, mm	343	425
Growing season mean air temperature, °C	14.5	14.3
Pedological indices		
Soil type	Calcaric Luvisol	
Soil texture	loam	
Sand, %	26.1	
Silt, %	48.4	
Clay, %	25.5	
pH_KCl_	7.42	
Soil organic matter, g kg^−1^	13.8	
N_total_, g kg^−1^	1.13	
Mobile P_2_O_5_, mg kg^−1^	152	
Mobile K_2_O, mg kg^−1^	161	

Note. SRC—standard rate of climate; growing seasons from 4 to 9 months.

**Table 5:** Crop rotation and harvest in the experimental site.

Season	Main Crop	Year of Harvest	Average Grain Yield, t ha^−1^	Average Straw Yield, t ha^−1^
2017	Spring field peas (*Pisum sativum* L.)	2017	4.0	3.6
2017–2018	Winter rape (*Brassica napus* L.)	2018	3.2	8.0
2018–2019	Winter wheat (*Triticum aestivum* L.)	2019	4.5	5.4
2019–2020	Winter wheat (*Triticum aestivum* L.)	2020	6.5	7.8
2020–2021	Winter rape (*Brassica napus* L.)	2021	3.6	9.0
2021–2022	Winter wheat (*Triticum aestivum* L.)	2022	7.5	9.0

## Data Availability

The original contributions presented in this study are included in the article. Further inquiries can be directed to the corresponding authors.
